# Palatable Levocetirizine Dihydrochloride Solid Dispersed Fast-Dissolving Films: Formulation and *In Vitro* and *In Vivo* Characterization

**DOI:** 10.1155/2022/1552602

**Published:** 2022-11-28

**Authors:** Sana Saleh Al-Kubati, Muna Abdo Ahmed, Nasr A. Emad

**Affiliations:** Department of Pharmaceutics, Faculty of Pharmacy, Aden University, P.O. Box 5411, Maalla, Aden, Yemen

## Abstract

One of the most important issues for bitter-tasting drugs such as levocetirizine dihydrochloride (LCD) is the production of palatable dosage forms. LCD also has a delayed onset of action following oral administration. In this study, solid dispersed fast-dissolving films (FDFs) of LCD using the solvent casting method for oral application were prepared and evaluated. The FDF is composed of HPMC as the film forming polymer and different types of superdisintegrants (sodium starch glycolate, croscarmellose sodium, or crospovidone). FDF containing crospovidone showed the highest percentage release of the drug (100.54% ± 1.47 within 3 min.) and was chosen for fabricating into palatable solid dispersed FDFs using different ratios of gelatine. The results of Raman and FTIR revealed that the drug's crystalline structure has been disrupted, and the drug has intermolecular hydrogen bonds with gelatine. The solid dispersed FDF (LF-7), which contained the drug in the form of a 1 : 1 solid dispersion with gelatine, showed a rapid *in vitro* disintegration (25 seconds) and a burst release of the drug (99.22% ± 2.22 within one min). The in vivo studies were conducted on human participants and showed a significant (*p* < 0.05) reduction in disintegration time (9.43 ± 2.16 sec.) and higher taste masking ability of the solid dispersed FDF (LF-7) compared to the nonsolid dispersed FDF (LF-4). The stability studies indicated that the prepared FDF remained stable over three months. Overall, FDFs of levocetirizine dihydrochloride with a palatable and rapid onset of action were developed to relieve allergic symptoms.

## 1. Introduction

The oral route bears various advantages over others for drug administration with some restrictions that arise from administering solid oral dosage forms. Low bioavailability, delayed onset of action, difficulty in swallowing or chewing, and taste masking have led to the development of advanced dosage forms suitable for more patient compliance, such as fast-dissolving films (FDFs) [1, 2]. Oral fast-dissolving films are composed of hydrophilic polymers, which rapidly dissolve or disperse once it comes in contact with the buccal cavity or tongue. FDFs are instantly hydrated by the saliva, adhere to the oral mucosa, and subsequently release the drug for absorption through the highly vascularised oral mucosa. This transmucosal absorption results in rapid onset of action with local and systemic effects and bypasses the first-hepatic metabolism or degradation in the gastrointestinal tract, leading to improved bioavailability [3, 4]. In addition, the formulation of FDFs by using natural or synthetic polymers such as gelatine and hydroxypropyl methylcellulose (HPMC) with appropriate sweeteners and flavoring agents is sufficient to achieve good mouth feel and taste masking [5].

LCD is a selective, nonsedative, and long-active antihistamine drug with less side effects. It was found that in adults, with the administration of 5 mg of oral solution and tablets of LCD, the peak plasma concentrations were achieved in 0.5 and 0.9 h, respectively [6]. LCD has a bitter taste and is classified as the BCS-III class under the biopharmaceutical classification system [7]. Thus, many taste-masking techniques are adapted to reduce or eliminate bitterness by obscuring the unpleasant taste of drugs or preventing dissolved drugs from interacting with taste buds [8, 9]. Some taste-masking techniques were used in order to mask LCD bitter taste such as complexation with HP-*β*CD, solid dispersion with mannitol, and ion exchange resins [10–14]. It was reported that the bitter taste of the drugs could be masked by using solid dispersion with a suitable polymeric carrier [15]. The bitter substances are bound to an excipient or trapped in a particulate, reducing their concentration in the oral cavity and preventing their release.

Gelatine is a natural protein, with both positive and negative charged groups, hydroxyl groups, and hydrophobic groups. It has the ability to form complexes that are stabilized by electrostatic interactions, hydrogen bonds, and to a lesser extent by hydrophobic interactions [16]. Gelatine is used conventionally as an excipient in capsules and tablet formulations to ease swallowing and as a taste-masking agent. It has been reported that gelatine forms a gel on the surface of the tablets to mask the bitter taste of the drugs and, as a film forming agent, it rapidly dissolves, producing a smooth mouth feel [15].

In this study, authors aimed to develop solid dispersed fast-dissolving films of levocetirizine dihydrochloride by using gelatine as a dispersing agent for LCD and hydroxypropyl methylcellulose as a film forming agent. Furthermore, superdisintegrants, sweeteners, and flavoring agents were used to formulate a palatable LCD solid disperse FDFs, thereby achieving a fast onset of action to relieve the symptoms associated with allergic conditions.

## 2. Materials and Methods

### 2.1. Materials

Levocetirizine dihydrochloride (LCD) was obtained as a gift sample from Modern Pharmaceutical Company, Sana'a, Yemen; HPMC and propylene glycol were purchased from Sigma-Aldrich, UK; sodium starch glycolate (SSG), croscarmellose sodium (CCS), crospovidone (CP), and gelatine were purchased from SD Fine Chemicals, India; sucrose, vanilla, and citric acid were purchased from Loba Chemie, India. All chemicals used were of analytical grade.

### 2.2. Preparation of LCD Fast-Dissolving Films

Table 1 shows the compositions of the seven FDFs of LCD in which HPMC is used as the film-forming polymer, propylene glycol as the plasticizer, and three types of superdisintegrants at a concentration of 6% *w/w*: SSG, CCS, and CP. Citric acid stimulates the saliva, sucrose as a sweetener, and vanilla as a flavoring agent. The solvent casting method was used to prepare these films. HPMC was dispersed in 20 ml of hot deionized water and stirred with a magnetic stirrer until the gel was formed. For formulations LF-1, LF-2, LF-3, and LF-4, the drug is dissolved with the other excipients in deionized water using a magnetic stirrer. Then, the drug solution of each formula was added separately to the HPMC gel containing propylene glycol and thoroughly stirred for about 30 min to ensure a uniform distribution of the drug solution. Then, they were left aside to remove air bubbles and then cast into a Petri dish (5.5 cm in diameter). They were left to dry for 24 h and were carefully removed from the Petri dish, checked for any imperfections, and cut into 1 cm^2^ films as each film contained 5 mg of LCD.

The solid dispersions at weight ratios of 1 : 0, 1 : 0.75, and 1 : 1 (*w/w*) of LCD: gelatine, were obtained by the solvent evaporation method. The calculated amounts of LCD were added to the dissolved gelatine aqueous solutions and left to dry for 24 h at room temperature to obtain solid films, which were then crushed and dissolved in deionized water with the same excipients used for formulation LF-4. Then, the solid dispersed drug solutions were added to the HPMC gels and followed the same procedures as for preparing the FDFs to obtain the formulations LF-5, LF-6, and LF-7.

### 2.3. Characterization of the Solid Dispersed LCD with Gelatine

#### 2.3.1. Fourier Transform Infrared Spectroscopy

The FTIR spectra for pure LCD, gelatine, and the solid dispersions of LCD with gelatine at 1 : 0.5, 1 : 0.75, and 1 : 1 *w/w* were analysed by using an FTIR spectrophotometer (PerkinElmer Spectrum, version 10.6.2) over the wavelength number range of 4000–450 cm^−1^ to investigate if there was any interaction in solid-state between LCD and gelatine.

#### 2.3.2. Raman Spectroscopy

A portable Raman spectrometer has a wavelength of 785 nm with a resolution of 8–10 cm^−1^, and a laser-class of Mira P advanced class 3B was used to investigate the crystalline state of LCD. A few milligrams of the powder samples of pure drug, gelatine, and solid dispersions of LCD with gelatine at weight ratios of 1 : 0.5, 1 : 0.75, and 1 : 1 were closed in glass vials. Then, the spectra of the samples by the laser power of about 100 mW room temperature over the spectral range of 2300–400 cm^−1^ using Mira Cal P software were recorded.

### 2.4. Characterization of LCD Fast-Dissolving Films

#### 2.4.1. Thickness, Weight Variation, and Folding Endurance

The thickness of each formulated FDF was measured at nine different positions on the film (the centre and eight corners) using a micrometer screw gauge. The weight uniformity was determined by taking the individual weights of 10 randomly selected 1 cm^2^ films from each formulation using a calibrated digital balance. Folding endurance for each oral FDF was determined by folding at the same location repeatedly until it broke or folding up to 300 times without breaking [17].

#### 2.4.2. Surface pH Measurement

Three 1 cm^2^ films of each formulation were allowed to dissolve in 5 ml of saliva simulated phosphate buffer (pH 6.8) at room temperature, and the pH was measured by bringing a combined glass electrode into contact with the solution and allowing it to equilibrate for 1 min. [18].

#### 2.4.3. Drug Content Uniformity

The seven formulae were evaluated for drug content uniformity by dissolving 3 films of each formula using phosphate buffer solution (pH 6.8) in a 50 ml volumetric flask that were diluted to the mark with the buffer and filtered through a 0.45 *μ*m membrane filter. The drug absorbance was measured spectrophotometrically at a maximum wavelength of 232 nm against a blank [19]. The concentrations were calculated from the equation of the standard calibration curve of LCD (*y* = 0.0311*x*−0.0089, *R*^2^ = 0.9995).

#### 2.4.4. *In Vitro* Disintegration Time

One film of each formulation was put separately in a Petri dish containing 10 ml of deionized water and was agitated smoothly and continuously. The time at which the FDFs started to disintegrate or break was recorded in seconds as disintegration times [18].

#### 2.4.5. Moisture Absorption and Loss

Three films from each formula were weighed separately (*W*1) and kept inside a desiccator containing silica gel for measuring moisture loss. For moisture absorption, three accurately weighed films from each formula were placed in a desiccator containing a saturated solution of aluminium chloride and keeping the humidity inside it at 75 ± 5% RH. The samples were kept for three days, and then the films were reweighed (*W*2), and the moisture loss and uptake were calculated using the following equations, respectively [20].

(1) $$\begin{array}{c} \%\text{ Moisture Loss}=\frac{\left(W1-W2\right)}{W1}\times 100,\\ \%\text{ Moisture Absorption}=\frac{\left(W2-W1\right)}{W1}\times 100. \end{array}$$

#### 2.4.6. *In Vitro* Dissolution Studies

The dissolution studies of the drug from FDFs were carried out using the USP rotating paddle dissolution test type II apparatus. The films were attached to the bottom of the dissolution vessels centrally below the paddle as gelatine has an adhesive property. The dissolution medium consisted of 500 ml of simulated saliva phosphate buffer solution (pH 6.8) maintained at 37 ± 0.5°C and rotating at 50 rpm. Samples of 5 ml were withdrawn at predetermined time intervals of 1, 2, 3, 4, 5, 6, 7, 8, 9, 10, 15, and 30 min and were replaced with equal volumes of the fresh medium kept at the same temperature. The samples were filtered through a 0.45 *μ*m membrane filter and analysed using a UV spectrophotometer at (*λ*max 232 nm). At each time interval, the concentration and the amount of the drug dissolved were calculated using the equation of the standard calibration curve of LCD. The drug dissolution profiles were constructed by plotting the cumulative percentage of the drug dissolved versus time [19].

### 2.5. Statistical Analysis

All the above parameters were carried out in triplicate, and the results were expressed as mean values and standard deviations (±S.D).

### 2.6. Evaluation of Palatability and *In Vivo* Disintegration Time on Human Participants

The palatability, mouth feel, and *in-vivo* disintegration time were tested for pure drug powder and selected FDFs of LCD that exhibited a high percentage of the drug released in a short time, LF-4 and three FDFs that contained solid dispersions of LCD with gelatine (LF-5, LF-6, and F-7). The study was performed on six healthy human adult participants (two females and four males) whose ages ranged from 23 to 25 years. The study protocol was approved by the Ethics Research Committee of the Faculty of Medicine and Health Sciences, University of Aden (REC-118-2022). The study protocol was explained, and written informed consent was obtained from all participants in the studies. The participants were asked to administer a pure powder (5 mg) in their mouths to assess the degree of bitterness and register their scores as 0: not bitter, 1: slightly bitter, 2: bitter, 3: moderately bitter, and 4: strongly bitter. The mouth was thoroughly rinsed with water, and a time of 30 min was kept between each trial. The same participants were asked to apply the same procedures by the administration of the selected films. The time required in seconds for the starting disintegration of the films and the irritation effect, if any, were recorded [21]. The results of palatability were analysed for statistical significance at *p* < 0.05 by using a one-paired Student's *t*-test.

### 2.7. Stability Studies

The stability studies were performed as per ICH guidelines for the selected formula LF-7. The films were wrapped in aluminium foil, packaged in an amber screw glass bottle, and stored in a desiccator at 45°C with a humidity of 75 ± 5% RH for three months. The films were evaluated after one and three months for weight, thickness, disintegration time, dug content, and dissolution studies [22].

## 3. Results and Discussion

Seven formulations were made using 3% *w/w* of HPMC as a film-forming polymer, and each film dose contained 5 mg of LCD. Then, the formulation that showed the high percentage of cumulative drug release in a short time, LF-4, was subjected to modification in order to obtain palatable FDFs. In this study, a solid dispersion technique was used, utilizing gelatine as a dispersing agent for LCD at a weight ratio (*w/w*) of drug to gelatine of 1 : 0.5, 1 : 0.75, and 1 : 1. The addition of a greater amount of gelatine results in a reduction in the quality of the films. So, the addition of the drug in the form of solid dispersion with gelatine to the compositions of the formulation LF-4 at these weight ratios was suitable to produce uniform, smooth, and flexible films.

### 3.1. Characterization of the Solid Dispersed LCD with Gelatine

#### 3.1.1. Fourier Transform Infrared Spectroscopy

**Figure 1:**
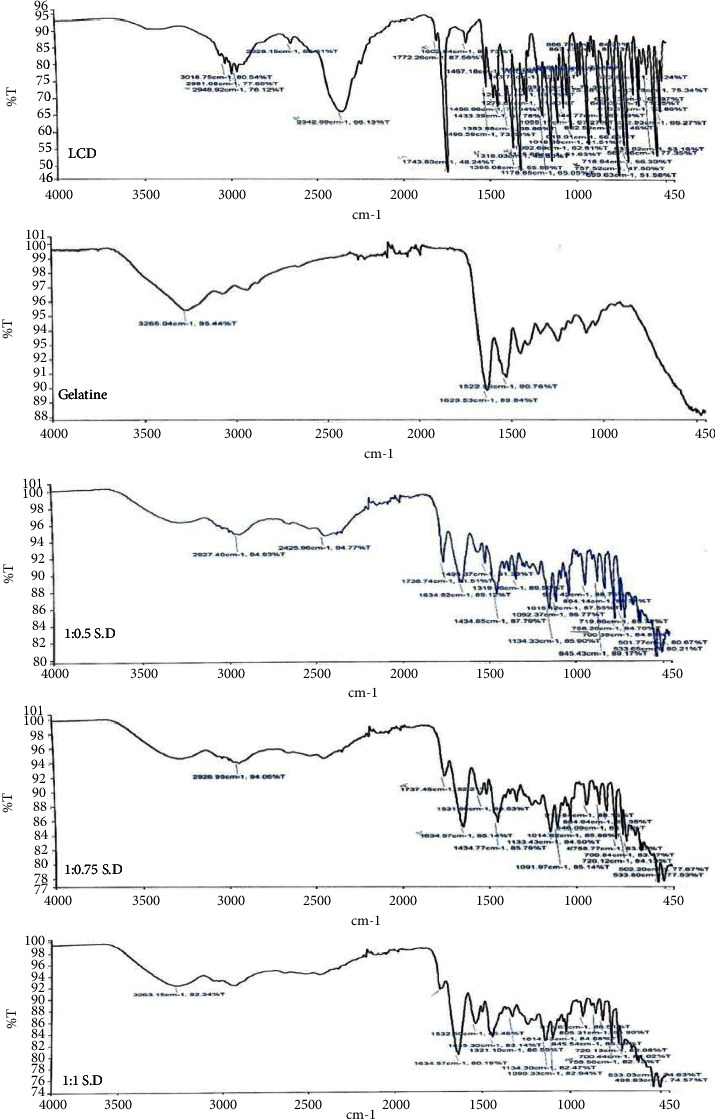
FTIR of levocetirizine dihydrochloride (LCD), gelatine, and the solid dispersions (S.D) of levocetirizine dihydrochloride with gelatine at weight ratios of 1 : 0.5, 1 : 0.75, and 1 : 1.

Figure 1(a) shows the FTIR spectra of pure LCD, which has characteristic bands at 3018.75–2948.92 cm^−1^ due to aliphatic and aromatic C-H stretching vibrations, a very strong absorption band at 2342.99 cm^−1^ due to carboxylic acid OH stretching vibration, a C = O stretching vibration at 1743.80 cm^−1^ and at 1602.84 cm^−1^ assigned to the stretching vibration of phenyl nucleus skeletal [23, 24]. In addition, the absorption bands were at 1318.03 cm^−1^, 1135.69 cm^−1^, and 757.52 cm^−1^ due to stretching vibrations of C-N, C-O, and C-Cl, respectively [6]. Gelatine consists entirely of amino acids joined together by amide linkages to form a linear polypeptide [5]. The FTIR spectra of gelatine (Figure 1(b)) shows a broad band at 3265.04 cm^−1^ attributed to NH and OH stretching vibrations, an absorption band at 1629.58 cm^−1^ due to the stretching vibration of amide carbonyl in amide I and at 1522.99 cm^−1^ due to NH and CN vibration of groups in amide in II [16]. As shown in figure (Figures 1(c)–1(e)), the peaks at regions 3018.75–2948.92 cm^−1^ are reduced in intensities in both 1 : 1.05 and 1 : 0.75 solid dispersions, while absence in 1 : 1 solid dispersion and the appearance of a single absorption broad band at 3263.15 cm^−1^. The strong broad band at 2342.99 cm^−1^ approximately disappeared. The intense sharp peak at 1743.80 cm^−1^ was greatly reduced in intensity and sharpness in the cases of 1 : 0.5 and 1 : 0.75 solid dispersions and was not recognized in 1 : 1 solid dispersion. On the other hand, the peak at 1602.84 cm^−1^ of LCD shifted with an increase in the height as the amount of gelatine increased in the solid dispersions. These findings reveal the presence of intermolecular hydrogen bonds between LCD and gelatine [25, 26].

#### 3.1.2. Raman Spectroscopy

Raman spectroscopy is an easy, rapid, and useful analytical technique for the investigation of the crystal forms of pharmaceutical compounds and excipients [27]. The differences can be observed between Raman spectra from different crystal forms of a compound or between crystalline and amorphous forms of pharmaceutical compounds [28]. Raman spectroscopy can be utilized to monitor the structural phase transition of a drug from crystalline to amorphous as it dissolves in the polymer [29]and for the assessment of the drug's solid-state physical stability and recrystallization kinetics during various storage conditions [30].

**Figure 2:**
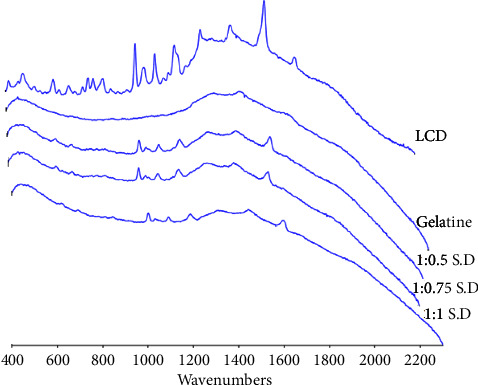
Raman spectroscopy of levocetirizine dihydrochloride (LCD), gelatine, and the solid dispersions (S.D) of levocetirizine dihydrochloride with gelatine at weight ratios of 1 : 0.5, 1 : 0.75, and 1 : 1.

The Raman spectroscopy of LCD, gelatine, and solid dispersions of them at 1 : 0.5, 1 : 0.75, and 1 : 1 *w/w* is depicted in Figure 2. The observed Raman spectra of LCD confirms its crystalline nature, as evidenced by the number of distinctive and intense peaks situated between wavenumbers 2300–400. However, Raman spectra of gelatine showed diffused peaks, indicating its amorphous nature. As shown in Raman spectra of solid dispersions, the characteristic peaks of LCD are nearly absent in solid dispersions, and they show the gelatine pattern, particularly at a solid dispersion of 1 : 1. The crystalline structure of the drug is disrupted when dissolved in gelatine, and the loss of crystallinity indicates that there are intermolecular interactions between the drug and gelatine [29,31].

### 3.2. Characterization of LCD Fast Dissolving Films

The results of the evaluated parameters of the prepared FDFs of LCD are presented in Table 2.

#### 3.2.1. Thickness, Weight Variation, and Folding Endurance

The prepared FDFs were smooth on both sides and transparent (LF-1, LF-2, LF-3, and LF-4), whereas the gelatine-containing formulations were opaque (LF-5, LF-6, and LF-7). No cracks or air bubbles were observed. They were peel-able, nonsticky, and flexible. The average weights of all formulae varied between 21.77 ± 1.81–30.29 ± 2.01 mg. The average thickness of all FDFs was between 20.78 ± 1.80 *μ*m–27.46 ± 1.09 *μ*m. It was observed that the weight and thickness were uniform within each formulation.

The folding endurance values for all the prepared LCD FDFs were found to be more than 300 times, which gives a good indication of the flexibility and the ability of the films to withstand rupturing.

#### 3.2.2. Surface pH Measurement

The surface pH of the FDFs of each formula was investigated in order to predict the possibility of any side effects occurring due to the change in the pH of the oral saliva, as acidic or alkaline pH may cause irritation to the oral mucosa. The formulations exhibited a surface pH between 5.69 ± 0.18 and 5.96 ± 0.12, which was close to the salivary pH range (6.5–6.8) [32].

#### 3.2.3. Drug Content Uniformity

The results of the percentages of drug content uniformity were between 96.06 ± 1.40 and 102.68 ± 1.31 which were within the acceptable range in all prepared formulae.

#### 3.2.4. *In Vitro* Disintegration Time

**Figure 3:**
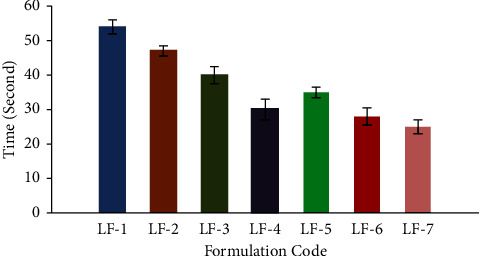
*In-vitro* disintegration time in seconds of fast-dissolving films of levocetirizine dihydrochloride (LF-1-LF-7).

From the results obtained in Table 2 and Figure 3, it could be seen that all the prepared FDFs were rapidly disintegrated in less than one min, but LF-7 containing gelatine and CP exhibited shorter disintegration times of 25 ± 2.00 sec compared to the others and hence was expected to obtain a fast release of the drug.

#### 3.2.5. Moisture Absorption and Loss

Moisture absorption and loss were measured to ensure the films' integrity under humid and dry conditions, respectively. The loss of water content may contribute to the brittleness of the films, whereas the gain of water may cause sticky films [5]. The moisture uptake results for the prepared films ranged between 5.11% ± 0.13 and 6.88% ± 0.10, and moisture loss results ranged between 4.17 ± 0.07% and 6.90 ± 0.14 which could be attributed to the presence of hydrophilic polymers [33].

#### 3.2.6. *In Vitro* Dissolution Studies

**Figure 4:**
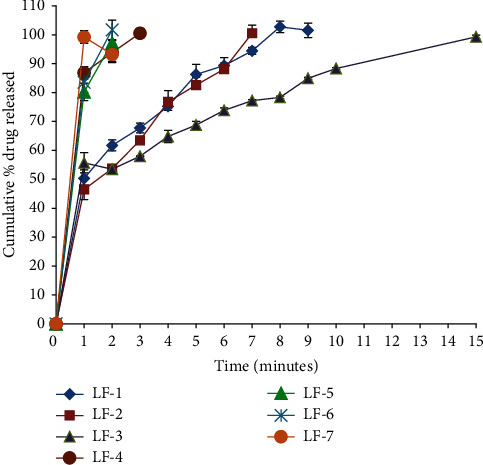
*In vitro* release profiles of levocetirizine dihydrochloride from the prepared fast-dissolving films (LF1-LF-7).

*In vitro* release studies for all of the prepared formulations were determined using saliva simulated fluid as a release medium (pH 6.8). The cumulative percentage release of LCD per minute is depicted in Figure 4. It was found that all the prepared formulae exhibited a high percentage of the drug released after one min (Table 2), which could be attributed to the use of HPMC, which has a burst effect just presenting the films in the dissolution medium [34]. The time required to release approximately 100% of the LCD, on the other hand, varied depending on the composition of the FDFs. Films formulated without the superdisintegrant LF-1 showed 101.51% ± 2.51 within 9 min. Formulae that contained three types of superdisintegrants, LF-2, LF-3, and LF-4, showed percentages of the drug released of 100.62% ± 2.82, 99.26% ± 0.63, and 100.54% ± 1.47 within 7, 15, and 3 min, respectively, which may be due to the difference in their mechanisms of hydration capacity that facilitate the disintegration of the films either by absorbing water rapidly followed by swelling (SSG), wicking and swelling (CCS), or by capillary action (CP). It was found that FDFs containing SSG and CCS exhibited longer times in the drug released compared with FDFs containing CP, which may be attributed to the tendency of SSG and CCS to swell and may form viscous gel layers around the films when fully hydrated that act as barriers for further rapid release of the drug. In contrast to SSG and CCS, CP is highly porous in nature, resulting in the production of porous films that exhibit virtually no tendency toward gel formation, even when using in high concentrations [35–37]. Gelatine films produce a smooth mouthfeel and rapidly dissolve. This is clearly seen when FDFs are fabricated by using solid dispersions of the drug with gelatine. Furthermore, increasing the ratio of gelatine to LCD from 0.5 in LF-5 to 1 in LF-7 increases the porosity of the film [35–37], which subsequently results in the fast release of LCD (LF-5 97.22% ± 1.40, LF-6 101.71% ± 3.00 within 2 min, and LF-7 99.22 ± 2.22 within one minute).

### 3.3. Evaluation of Palatability and *In Vivo* Disintegration Time on Human Participants

**Figure 5:**
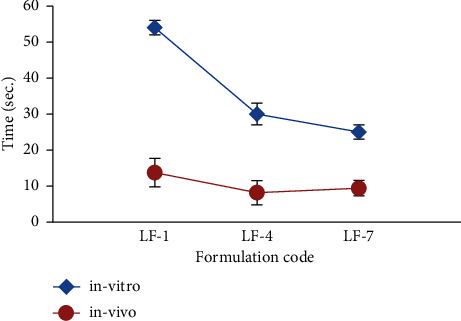
Comparison between *in vitro* and *in vivo* disintegration time of the selected fast-dissolving films of levocetirizine dihydrochloride LF-1, LF-4, and LF-7).

Table 3 depicts the mean scores of the selected formulations for the taste masking ability of the bitter taste of LCD. All the selected FDFs contained sucrose and vanilla. The drug powder has a strong bitter taste, as indicated by the participants who gave it a mean score of 4. However, FDFs containing LCD as solid dispersions with gelatine exhibited significantly lower scores for taste masking ability (LF-5 and LF-6; *p* < 0.05) and (LF-7; *p* < 0.001) compared to LF-4. The success of producing palatable FDFs of LCD was made more pronounced by the incorporation of LCD as a solid dispersion with gelatine at a weight ratio of 1 : 1 into FDFs, which indicated that G-protein coupled receptors are shielded from the effects of LCD since the latter is dispersed uniformly across the gelatine crust [38]. In addition, effective taste masking can also be attributed to the flavours. It was observed that rapid *in-vivo* disintegration times of 13.71 ± 3.96, 8.14 ± 3.38, and 9.43 ± 2.16 sec (Table 3) compared with the *in-vitro* disintegration times of 54 ± 2.00, 30 ± 2.00, and 25 ± 2.00 sec for LF-1, LF-4, and LF-7, respectively (Figure 5). The fast in vivo disintegration may have been facilitated by the tongue's pressing movement against the palate to retain film attachment. Another possible explanation for the shortened in vivo disintegration time is the incorporation of citric acid into the FDFs, which promoted saliva secretion in the buccal cavity and contributed to the faster disintegration of the FDFs [12,39].

### 3.4. Stability Studies

The results of stability studies of the selected palatable FDFs of LCD LF-7 are presented in Table 4. During the storage periods, there were no differences in drug content, disintegration time, or drug release. The films also showed satisfactory flexibility, weight variation, and thickness. The results revealed that LF-7 was stable during the storage periods for the three months at 45°C and a humidity of 75 ± 5% RH [40].

## 4. Conclusion

In conclusion, levocetirizine dihydrochloride was incorporated as solid dispersions with gelatine into HPMC fast-dissolving films. At a 1 : 1 weight ratio, the films disintegrated rapidly in less than 30 seconds, approximately completely releasing the drug within one minute and remaining stable over the storage period. The bitter taste was masked effectively by using the solid dispersion method. So, a palatable and rapid onset of action FDFs of levocetirizine dihydrochloride was obtained to relieve the allergic symptoms of allergic rhinitis and upper respiratory tract inflammation.

## Figures and Tables

**Table 1:** Compositions of fast-dissolving films of levocetirizine dihydrochloride.

Ingredient	LF-1	LF-2	LF-3	LF-4	LF-5	LF-6	LF-7
LCD (mg)	120	120	120	120	—	—	—
LCD: gelatine S.D^*∗*^	—	—	—	—	1 : 0.5	1 : 0.75	1 : 1
HPMC (%)	3	3	3	3	3	3	3
SSG (%)	—	6	—	—	—	—	—
CCS (%)	—	—	6	—	—	—	—
CP (%)	—	—	—	6	6	6	6
PG (ml)	0.2	0.2	0.2	0.2	0.2	0.2	0.2
Sucrose (mg)	34	34	34	34	34	34	34
Vanilla (mg)	14	14	14	14	14	14	14
Citric acid (mg)	13	13	13	13	13	13	13
Water (ml)	20	20	20	20	20	20	20

S.D^*∗*^: solid dispersion.

**Table 2:** Evaluation parameters of the prepared fast-dissolving films of levocetirizine dihydrochloride (LF1-1-LF-7).

Evaluation parameter	Formulation code						
	LF-1	LF-2	LF-3	LF-4	LF-5	LF-6	LF7
Appearance	Transparent	Transparent	Transparent	Transparent	Opaque	Opaque	Opaque
Weight variation (mg)	21.77	23.81	24.21	25.91	28.43	29.02	30.29
	±1.81	±2.20	±2.80	±2.60	±2.51	±3.01	±2.01
Thickness (*μ*m)	20.78	23.22	23.89	23.56	24.44	25.63	27.46
	±1.80	±1.73	±1.96	±2.28	±1.58	±1.69	±1.09
Folding endurance	>300	>300	>300	>300	>300	>300	>300
Surface pH	5.80	5.96	5.69	5.84	5.94	5.91	5.85
	±0.11	±0.12	±0.18	±0.20	±0.10	±0.15	±0.15
Drug content uniformity (%)	99.72	97.67	102.68 ± 1.31	96.06	99.73	98.21	98.31
	±2.70	±1.23		±1.40	±1.20	±0.8	±1.02
Moisture uptake (%)	5.71	6.76	5.33	5.11	6.20	6.62	6.88
	±0.06	±0.16	±0.15	±0.13	±0.09	±0.10	±0.10
Moisture loss (%)	5.88	6.54	6.90	4.17	5.77	5.38	5.20
	±0.071	±0.12	±0.14	±0.07	±0.11	±0.13	±0.12
*In vitro* disintegration (Sec.)	54	47	40	30	35	28	25
	±2.00	±1.50	±2.50	±3.00	±1.5	±2.5	±2.00
*In vitro* drug released (%) after one min.	50.29	46.49	55.71	86.82	80.4	83.52	99.22
	±2.97	±3.47	±3.05	±2.20	±3.30	±3.40	±2.20

(*n* = 3; mean ± SD).

**Table 3:** Score mean for evaluation of palatability and *in vivo* disintegration time on human participants of the selected fast-dissolving films of levocetirizine dihydrochloride.

Formulation code	*V*-1	*V*-2	*V*-3	*V*-4	*V*-5	*V*-6	Score mean	^*∗*^ * In vivo* disintegration time (sec.)
	Score							
Drug powder	4	4	4	4	4	4	4.0	—
LF-4	2	3	2	4	2	4	^#*∗∗*^2.8	8.14 ± 3.38
LF-5	0	2	1	1	3	1	^#^1.3	—
LF-6	0	2	1	1	2	0	^#^1.0	—
LF-7	0	1	0	0	1	0	^*∗∗*^0.3	9.43 ± 2.16

^*∗*^(*n* = 3; mean ± SD), *V*: volunteer, scores: 0-non bitter, 1-slightly bitter, 2-bitter, 3-moderate bitter, and 4-strong bitter (^#^*p* < 0.05, ^*∗∗*^*p* < 0.001).

**Table 4:** Stability studies of the optimized fast-dissolving films (LF-7) of levocetirizine dihydrochloride.

Evaluation parameter	Fresh	One month	Three months
Weight variation (mg)	30.29	30.09	30.20
	±2.01	±2.99	±2.96
Thickness (*μ*m)	27.46	26.66	27.67
	±1.09	±2.16	±3.15
*In-vitro* disintegration (sec.)	25	21	35
	±2.00	±3.41	±1.35
Drug content	98.31	99.01	95.24
Uniformity (%)	±1.02	±0.055	±0.048
*In-vitro* drug release (%)	99.22	101.82	97.12
	±2.20	±0.65	±1.95

(*n* = 3; mean ± SD).

## Data Availability

The data supporting the conclusion of this study will be provided by the corresponding author upon request.
